# Five-year single-centre experience of carcinoma of the oesophagus from Blantyre, Malawi

**DOI:** 10.1136/bmjgast-2018-000232

**Published:** 2018-10-15

**Authors:** John David Chetwood, Peter J Finch, Anstead Kankwatira, Jane Mallewa, Melita A Gordon, Leo Masamba

**Affiliations:** 1 Department of Medicine, College of Medicine, University of Malawi, Blantyre, Malawi; 2 Malawi-Liverpool-Wellcome Trust Major Overseas Programme, Blantyre, Malawi; 3 Institute of Infection and Global Health, University of Liverpool, Liverpool, UK; 4 World Gastroenterology Organisation International Training Centre, Blantyre, Malawi; 5 Department of Medicine, Queen Elizabeth Central Hospital, Blantyre, Malawi

**Keywords:** oesophageal cancer, oesophageal disease, oesophageal lesions, cancer epidemiology, carcinogenesis

## Abstract

**Background:**

Oesophageal squamous cell carcinoma (OSCC) is increasing worldwide and has an exceptionally high prevalence in certain distinct geographical locations such as the African oesophageal SCC corridor. Despite this, there is a paucity of evidence to characterise the disease particularly in the Malawian context.

**Method:**

We retrospectively audited our endoscopy database over 5 years, including for patient demographics, endoscopy findings, therapeutic intervention and recommendations for treatment.

**Results:**

1586 patients with oesophageal cancer were identified from a total of 5882 endoscopy records from 2013 to 2017. Our cohort showed a larger proportion of oesophageal cancers found higher in the oesophagus compared with other African studies and a female preponderance in this upper-oesophagus disease subset though a male preponderance overall. 39% of patients with oesophageal cancer underwent bougie dilatation and 11% underwent palliative stent placement, which likely reflects local availability of resources.

**Conclusion:**

This study validates the observation that OSCC predominates in sub-Saharan Africa in Malawi over other forms of oesophageal carcinoma, though our cohort appears to have subtly distinct demographics and disease-specific data. This highlights the need to prioritise preventative and therapeutic strategies for OSCC in this and similar settings.

**Summary box**

**What is already known about this subject?**

- Oesophageal squamous cell carcinoma (OSCC) is the most common type of oesophageal cancer worldwide and is extremely prevalent in certain delineated locations such as across the African Rift Valley.
- Malawi has the highest incidence of OSCC in the world.
- Despite this, there is a relative lack of data in the African and particularly Malawian setting including characterising endoscopic findings and used treatment.

**What are the new findings?**

- This is the first study of its type to characterise endoscopic data for one of the principal endoscopy sites in Malawi.
- This confirms the large predominance of OSCC as a diagnostic finding at endoscopy, validating the emerging importance that is being placed on this disease.
- Our population cohort had a predominance of OSCC located in the upper oesophagus compared with Africa-wide data and a female predominance in this upper OSCC cohort.
- Likely due to resource availability, bougie dilatation of OSCC predominated as a palliative measure over stent placement and was safe in this setting.

**How might it impact on clinical practice in the foreseeable future?**

- This validates the increasing importance that OSCC is having on Malawian and international health agendas.
- This is the first study of its type to characterise this cohort including their demographics and disease characteristics.
- Bougie dilatation is common and safe in the Malawian setting for OSCC.

## Introduction

There is wide geographical variation in the incidence of many cancers which may reflect aetiological risk factors; in particular in the case of oesophageal squamous cell carcinoma (OSCC), there is a strikingly high incidence in the African Rift valley, the so-called African oesophageal SCC corridor^1^ extending from Sudan to the Eastern Cape Province of South Africa and including Malawi. Malawi ranks number one in the world for the incidence of oesophageal cancer with a weighted age standardised rate (ASR) of 24.2 (world ASR=5.9).^2^ Malawi also ranks as one of the poorest countries in the world, ranked second lowest in income (after Burundi) in the world with a per capita gross national income of US$320 per annum in 2017^3^—which will likely be affected in 2018 by the anticipated erratic rainfall and armyworm infestation on agriculture production.

Unfortunately, OSCC has particularly poor outcomes in low-income settings, where the disease often presents too late for therapeutic intervention, with only 13% of patients in sub-Saharan Africa being surgical candidates and far fewer being able to have access to or afford such a treatment.^4^ As such a diagnosis is generally terminal, OSCC in such settings has been neglected from research agendas and it is widely accepted more work is needed in this area to further understand the risks and disease development in this context.^5 6^

The endoscopy unit at the Queen Elizabeth Central Hospital in Blantyre has a single procedure room and is a World Gastroenterology Organisation International Endoscopy Training Centre forming the centre of a hub-and-spoke endoscopy training programme for the three other central hospitals in the country. It performs around 1200 upper gastrointestinal endoscopies per annum, of which 300 are therapeutic—mainly dilatation or stenting of SCC and banding of oesophageal varices. All cases are recorded in a bespoke electronic recording system. In view of the enormous burden of oesophageal SCC that we see here, we audited the data from the endoscopy unit to analyse clinical and endoscopic features of SCC presenting over 5 years.

## Methods

The endoscopy computer database is a Microsoft Access non-relational database recording patient demographics, endoscopy findings, therapeutic intervention and recommendations for treatment. All endoscopy records held in the departmental computer database for a 5-year period (2013–2017) were examined and any associated histology reports sought. Repeat endoscopies on the same individual were eliminated. Surgical and Oncology databases were also interrogated for supplementary routine clinical details of patients with SCC during the same time period, but direct linkage was impossible in the absence of hospital numbers, consistent spelling of names or date of birth (hospital numbers are not assigned, many patients do not know their date of birth, or even age, and spelling of names is variable). The data were analysed using Statistical Package for Statistical Sciences (SPSS/PC+ V.3.1) to undertake one-way analysis of variance and regression analysis between the age and gender of patients stratified according to the distance of the tumour from the teeth/gums (upper <25 cm, mid 25–29.9 cm, lower 30–34.9 cm and GOJ (gastro-oesophageal junction) ≥35 cm) using Student-Newman-Keuls (SNK) multiple range testing for significant differences in sorted means depending on the number of steps between the two means being tested, and χ^2^ test for gender differences stratified by distance, with Kendall’s Tau C as a measure of association for ordinal variables.

## Results

Of a total of 5882 endoscopy records, 1586 patients (27%) with an endoscopic diagnosis of oesophageal cancer were identified, 68% of procedures carried out by four of the authors (PJF, AK, JM, LM). The youngest patient was 17 years old and the oldest 100 years, median age 55 years. The presenting symptom was dysphagia in 84% of cases.

Results are summarised in table 1.

**Table 1:** Oesophageal cancer data stratified by site of tumour (upper <25 cm, mid 25–29.9 cm, lower 30–34.9 cm and GOJ ≥35 cm)

Site	Upper	Mid	Lower	GOJ	All
number at each site	349	449	488	300	1586
percent distribution at each site	22%	28%	31%	19%	100%
Age at each site					
Median	49	52	52	55	55
IQR	24	22	23	21	23
Minimum age	22	18	17	24	17
Maximum age	87	95	94	100	100
Female	186	205	202	123	716
% Female	53.3%	45.7%	41.4%	41.0%	45.1%
Histology at each site					
Squamous carcinoma	39	51	68	33	191
Undifferentiated carcinoma		1	2		3
Squamous dysplasia	4	12	6	5	27
Adenocarcinoma				6	6
Non-malignant	4	8	16	8	36
All	47	72	92	52	263

Statistically distinct identities indicated by the box outlines.

GOJ, gastro-oesophageal junction.

Analysis of variance showed highly significant differences in the age of patients stratified according to the site of the tumour (F_3,1582_=6.7, p<0.001), and SNK range testing revealed this was due to the ‘Upper’ oesophagus group being significantly (p<0.05) different from all the other groups which were statistically indistinguishable—the identity difference is indicated in table 1 by the boxes around the median ages for each site group. Likewise, there were highly significant differences in the gender of patients stratified according to the site of the tumour (F_3,1582_=4.8, p<0.01) and SNK range testing revealed this was due to the ‘Upper’ oesophagus having more women than all the other groups with the same identity differences as for age. Multiple regression analysis revealed a highly significant association between age and gender, and site: F_2,1583_=14.6, p<0.001 with a positive association between age and distance of the cancer down the oesophagus and more men with increasing distance down the oesophagus. Analysis of variance showed no gender differences when stratified by age: F_1,1584_=0.28, p=0.6.

Chi-squared analysis confirmed significant gender differences stratified by site of the tumour (χ^2^=14.3, *df* 3, p<0.01), and Kendall’s Tau C confirmed the female gender was associated with higher tumours (τ_c_=−0.10, p<0.001), 53.3% women in the ‘Upper’ oesophagus group and 41.0% in the ‘GOJ’ group.

A total of 263 histology reports were found. Most patients underwent biopsy at the time of endoscopy when it was technically feasible, but unfortunately, histology was only available for a limited proportion of the 5-year period in the Ministry of Health due to staff shortages and lack of reagents for staining and so on. When a histologist was available for reporting, patients were asked to make a contribution (~US$14) to fund the reagents and many chose not to. Likewise, at times a histology report was only available in the private sector at a cost of around US$41. The vast majority (83%, n=218) of tumours were shown to be due to squamous cancer/dysplasia and only 2% (n=6) due to adenocarcinoma. A number of biopsies (14%, n=36) showed non-malignant pathology (ulceration, slough, missed tumour etc), although the endoscopic diagnosis was clearly that of a tumour.

Thirty-nine per cent (620 patients) underwent bougie dilatation of their tumour for symptom relief, 11% (179 patients) had placement of a self-expanding metal stent (only sporadically available in our hospital), and one patient had alcohol injection of the tumour for debulking. Two perforations were identified after bougie dilatation and were managed conservatively. One per cent (17 patients) underwent an Ivor Lewis oesophagectomy with end-to-end anastomosis and 1% (22 patients) had palliative gastrostomy tubes inserted. Seventeen per cent (274 patients) received chemotherapy. Radiotherapy is not available at present anywhere in the country. We have no outcome data for any of these patients, in the absence of a Hospital Records System.

## Discussion

Lamentably, data on OSCC in sub-Saharan Africa are scarce and this is particularly true in Malawi which has the highest incidence of OSCC in the world.^2^ This study validates the observation that OSCC predominates in sub-Saharan Africa in Malawi over other forms of oesophageal carcinoma, the mechanism for which is still debated. One previous study has prospectively evaluated oesophageal carcinoma in this context,^7^ but it recruited much smaller numbers over a smaller period (143 patients over 9 months vs 1586 patients over 60 months) and was primarily aimed at following up expandable oesophageal stent placement rather than a descriptive assessment of all oesophageal cancers.

Compared with Africa-wide data, our population was similar though had a predominance of tumours anatomically located in the upper oesophagus (22% vs <20% for oesophageal cancer in a previous estimated prevalence study across Africa in 2012).^8^ Otherwise, the anatomical distribution seemed broadly similar (28% in the mid-oesophagus for ours vs 30%–70% in Africa in 2012,^8^ 50% in the lower oesophagus/GOJ vs 20%–50% in Africa in 2012).^8^

We noted a female preponderance in the upper third of the oesophagus, but a male preponderance overall which accords with previous Africa-wide data in 2012^8^ (though there ares heterogeneous demographic data across the continent).

The relatively young age of patients with OSCC at diagnosis compared with other high-incidence locations is consistent with other Africa-wide data; for example, 8% of OSCC cases in the Bomet district of West Kenya were under 30 years of age at diagnosis.^9^

Although smoking and alcohol play a prominent role in a higher-income context, these are unlikely to be causative for the observed prevalence in the high-incidence (low-resource) areas.^10 11^ First exposure to these risk factors does not reflect the observed OSCC disease prevalence; there are often similar rates of OSCC in men and women despite significantly different exposures to smoking and alcohol.^12^ Furthermore, exposure to these risk factors are not prominent practices in other high-prevalence areas such as China and repeatedly been shown not to be a major risk factor in OSCC development.^10^

There are many putative mechanisms for the observed high OSCC prevalence in this context, and a compelling potential cause includes fumonisin exposure—a mutagenic mycotoxin found on maize and associated with high OSCC rates.^13–16^ In the Malawian context, this may be driven by the cultural and financial reliance of maize as the predominant dietary constituent, as well as changes in traditional methods of storage of maize—from nkhokwe (well-ventilated grain silos on stilts) to indoor storage because of security from theft driven by extreme poverty—which facilitates fungal growth. Over-reliance on maize on a stable may also cause micronutrient deficiencies particularly due to the cooking methods in this context (though this is controversial)^8^ and due to silica contamination.^17^

However, other potential reasons include thermal damage to oesophageal mucosa (by hot drinks such as tea, but also the hot maize porridge ‘phala’; the young age of consumption and exposure may explain the young presentation of OSCC) as well as micronutrient including selenium deficiencies in an estimated 80% of the Malawian population due to poor crop uptake of selenium in the low pH soils,^18^ as well as exposure to polycyclic aromatic hydrocarbons from cook fire smoke in the home (particularly when lower socioeconomic groups are forced to use poorly combustible fuels due to financial constraints).^19 20^ Ultimately, the causative mechanism has not been proven and ultimately remains unclear.

As risk factors may disproportionately affect OSCC risk in different parts of the oesophageal mucosa,^21^ the female and younger individual preponderance in the ‘Upper’ oesophagus group in our setting may be attributable to different behaviours and therefore exposures (such as thermal damage) based on gender and age. However, this is speculative as it may also reflect a different underlying population, and therefore remains to be confirmed.

Though palliative stenting has good efficacy in our setting (<4% complication rate and a median survival of 210 days),^7^ the cost is unfortunately prohibitive for the health service and for patients, and as such, bougie dilation is often undertaken. There is a paucity of evidence of long-term outcomes for this procedure though itseems effective in other settings^22^ and carries a risk of malignant perforation.^23^

The strengths of these data include the large patient numbers and the long period the audit was undertaken, which enabled the detection of a distinct group of patients who have a higher risk of suffering from tumours higher in the oesophageal tract (ie, younger age and female). However, though this institution serves a large area, this study was undertaken at a single site and was unable to provide histological confirmation in the majority of cases given local funding constraints.

## Conclusion

We present retrospective endoscopic data of oesophageal cancers in the Malawian context, which validate the predominance of OSCC in this context. We assert this study demonstrates the need for prioritisation of OSCC on the national health agenda, including consideration of characterising the causative mechanisms, promoting preventative strategies and a cost-effectiveness assessment for a radiotherapy facility. Preventative strategies could take the form of joint public health strategies, for example if cook fire is validated as a risk factor, there already exist strategies to reduce domestic exposure due to its pulmonary sequelae,^24^ and micronutrient deficiency correction would have benefits beyond OSCC.^25^ We look forward to further data in this context which further characterise the risk factors.
