# Correction: *Five-year single-centre experience of carcinoma of the oesophagus from Blantyre, Malawi*

**DOI:** 10.1136/bmjgast-2018-000232corr1

**Published:** 2018-12-30

Chetwood JD, Finch PJ, Kankwatira A, *et al*. Five-year single-centre experience of carcinoma of the oesophagus from Blantyre, Malawi. *BMJ Open Gastro* 2018;5:e000232. doi: 10.1136/bmjgast-2018-000232

This article has been corrected since it first published. Owing to the correction in Table 1. This is corrected in the latest version.

